# The impact of the COVID-19 lockdown on social and economic welfare in Uganda

**DOI:** 10.1186/s13690-024-01337-x

**Published:** 2024-08-06

**Authors:** David Musoke, Sarah Nalinya, Grace Biyinzika Lubega, Kevin Deane, Elizabeth Ekirapa-Kiracho, David McCoy

**Affiliations:** 1 Department of Disease Control and Environmental Health, School of Public Health, College of Health Sciences, Makerere University, Kampala, Uganda; 2 The Open University, Milton Keynes, UK; 3 Department of Health Policy, Planning and Management, School of Public Health, College of Health Sciences, Makerere University, Kampala, Uganda; 4 International Institute for Global Health, United Nations University, Kuala Lumpur, Malaysia

**Keywords:** COVID-19, Lockdown, Socio-economic impact, Pandemic response, Uganda

## Abstract

**Background:**

As a measure to slow down the transmission of Coronavirus disease (COVID-19), governments around the world placed their countries under various stringent lockdown measures. Uganda is one of the countries that had a strict lockdown in Africa. This qualitative study explored the social and economic impacts of the COVID-19 lockdown in both an urban (Kampala) and rural (Wakiso) setting in Central Uganda.

**Methods:**

The study used focus group discussions (FGDs), household interviews, and key informant interviews (KIIs). 14 FGDs were conducted among several stakeholders including community health workers, health professionals, and members of the community. 40 household interviews were conducted among low, middle, and high-income households, while 31 KIIs were held among policy makers, non-governmental organisations, and the private sector. Data were analysed thematically in NVivo 2020 (QSR International).

**Results:**

Findings from the study are presented under six themes: family disruption; abuse of children’s rights; disruption in education; food insecurity; impact on livelihoods; and violation of human rights. The study found that the COVID-19 lockdown led to family breakups, loss of family housing, as well as increased both caring responsibilities and gender-based violence especially towards females. Children’s welfare suffered through increased child labour, sexual exploitation, and early marriages. The extended closure of schools led to delayed educational milestones, poor adaptation to home-based learning, and increased school drop-out rates. Increased food insecurity led to changes in feeding patterns and reduced food varieties. Livelihoods were negatively affected hence people depleted their savings and capital. Unlawful detention and beating by law enforcement officers increased during the lockdown.

**Conclusion:**

Future pandemic planning needs to consider the consequences of lockdown on the social and economic wellbeing of communities hence put in place appropriate mitigation measures during and after the outbreak.

**Table:** 

**Text box 1. Contributions to literature**
• Several countries including Uganda instituted various stringent lockdown measures to control the spread of COVID-19 during various stages of the pandemic.
• There is limited evidence on the social and economic impacts of the COVID-19 lockdown in Uganda and other parts of the world.
• The COVID-19 lockdown led to vast social and economic impacts in Uganda. The social and economic wellbeing of communities should be considered during measures to respond to future outbreaks in Uganda.

## Introduction

The Coronavirus disease (COVID-19) pandemic started in Wuhan, China in late 2019 [1] and due to international travel, cases were seen in Africa as early as February 2020 [2]. As of 31st March 2024, 775,251,779 cases had been confirmed and 7,043,660 people had reportedly died due to COVID-19 globally [3]. Within the African region, 9,577,797 confirmed cases [3] and 175,354 deaths have been reported since the start of the pandemic as of 31st March 2024 [4]. COVID-19 is transmitted from person to person through droplets or aerosols, airborne and surface transmission [5]. In order to slow down the transmission of COVID-19, governments around the world placed their countries under a variety of stringent lockdown measures. Other measures to prevent further spread of the disease included the use of face masks, social distancing, hand hygiene (regular hand washing with soap or use of alcohol hand sanitizers), and respiratory hygiene (covering of the mouth when sneezing and coughing, as well as avoidance of touching eyes and nose) [6]. Evidence suggests that lockdowns were necessary to reduce the spread of the highly infectious virus and prevent health facilities from being overwhelmed [7, 8]. In addition, COVID-19 lockdowns were considered a necessary intervention to give public health professionals time to think, plan and act accordingly in the interest of global health [9, 10].

COVID-19 took most nations by surprise, and stringent measures in many countries may have been unavoidable. Whereas lockdowns have been documented as effective containment measures for highly transmissible infectious diseases, they are also known for negatively impacting economic growth and social welfare in the communities in which they have been instituted [11–13]. To date, studies globally have documented several impacts of the COVID-19 lockdowns including: reduced access to education, food and healthcare [14]; unemployment and reduced income returns [15]; mental illnesses such as stress, anxiety and depression [16]; and gender-based violence [17]. A recent study showed that the socio-economic impact of the COVID-19 pandemic was worse in low- and middle-income countries (LMICs) and vulnerable populations [18]. Despite this evidence, there is limited literature on the social and economic impacts of the COVID-19 lockdowns particularly in Uganda. The findings from this study could be used to inform responses to future disruptions related to COVID-19 or other public health threats regarding maximising the benefits of lockdown while minimising the negative impact on social and economic welfare among the population.

In Uganda, the government placed the country under public health restrictions including a national lockdown in early April 2020, 14 days after Uganda’s first COVID-19 case in March 2020. The national lockdown consisted of: closure of schools, bars, places of worship and work with an exception of essential services such as healthcare and food; a ban on public transport; and a nation-wide curfew (movements only allowed between 6:00 am to 7:00 pm) [19]. Social distancing, hand hygiene and mask wearing were also required in public spaces. A report by UNICEF in 2020 showed that these measures also slowed down economic activities and pushed many Ugandans into extreme poverty [13]. One recent study argued that the socio-economic impacts of COVID-19 in Uganda might have outweighed the positive health impacts of the control measures [20]. Other studies in Uganda have documented the inevitable disruptions to the socio-economic situation of vulnerable populations [21, 22]. Child abuse, sexual abuse, gender-based violence [23, 24], and setbacks in education [25] have also been reported. Furthermore, reduced purchasing power, depletion of savings, and food insecurity such as reduction in number of meals per day have also been documented [26].

Six months after declaration of the COVID-19 pandemic, the United Nations (UN) launched a framework for a socio-economic response to the pandemic. The framework was intended to counter the interruption of social services and economic breakdown of societies before the impacts of the pandemic became permanent [27]. The framework comprised 5 streams: (1) ensuring that essential health services are still available and protecting health systems; (2) helping people cope with adversity, through social protection and basic services; (3) protecting jobs, supporting small and medium-sized enterprises, and informal sector workers through economic response and recovery programmes; (4) guiding the necessary surge in fiscal and financial stimulus to make macroeconomic policies work for the most vulnerable and strengthening multilateral and regional responses; and (5) promoting social cohesion and investing in community-led resilience and response systems [27]. In this study, we explored the impact of COVID-19 lockdown measures on social and economic welfare in Kampala and Wakiso districts in Uganda.

## Methodology

### Study design and setting

This was a cross-sectional qualitative study employing a phenomenological approach and used three data collection techniques: focus group discussions (FGDs), key informant interviews (KIIs), and household interviews. The study was conducted in two settings: one rural and the other urban. Kasanje Town Council in Wakiso district represented the rural setting while Rubaga Division in Kampala district represented the urban setting. These sites were selected for involvement in the study because they were among the most affected in terms of incidence of COVID-19 in the two districts at the time [18, 19]. The major economic activity was agriculture in the rural setting and small-scale business in the urban setting. Kasanje Town Council, with a projected population of 46,042 people, had one public health facility, two government-aided health facilities, nine registered clinics, and 22 registered drug shops [28]. Rubaga Division, with a projected population of 427,300 people [29], had two government health facilities, 11 private-not for profit health facilities, and over 40 private health facilities [30]. More details about the study setting can be found in our earlier published paper [31].

### Study participants and data collection

The study conducted a total of 14 FGDs, 31 KIIs, and 40 household interviews. FGD and KII guides, and a non-structured questionnaire were used during data collection. Guiding questions explored participants’ views of the social and economic effects of the COVID-19 lockdown. Issues explored included effects of the lockdown on small and medium business, jobs and employment, as well as housing and family dynamics among others.

The study participants of both the FGDs and household interviews were recruited from ten rural zones in Kasanje Town Council, Wakiso District and seven urban zones in Rubaga Division, Kampala City. These zones in Wakiso and Kampala were purposively selected by the research team because they were largely comprised of informal settlements, as well as had a high population and overcrowded households which increased their vulnerability to COVID-19. They also had limited access to social services such as clean water, and a high proportion of people with informal jobs and businesses hence were more susceptible to the socio-economic consequences of the COVID-19 control measures. Participants of the household interviews consisted of individuals from low, middle and high socio-economic classes which provided a broad spectrum of experiences and insights. One member per household participated in the study, with preference given to the household head or their spouse. Participants of the interviews provided personal, household and community experiences of the COVID-19 control measures and their social and economic effects. The interviews were conducted in *Luganda*, the most widely used local language in the study districts and audio recorded.

Participants of the FGDs were purposively selected by local leaders and community mobilizers with guidance from the research team. They were selected to represent separate female youth, male youth, older female, older male, community health workers and community leader groups. In total, 4 male FGDs, 4 female FGDs, and 6 mixed FGDs were conducted. These FGDs were comprised of between 8 and 16 individuals and conducted within the community at a public location. Separate FGDs were conducted among health practitioners at their respective health facilities. The health practitioners were selected by the health facility in-charge to represent various departments. The FGDs were participatory and involved assigning participants to smaller groups of 3–4 people. The small groups then discussed and re-created the timeline of various COVID-19 prevention and control measures from March 2020 to July 2021. Thereafter, they discussed the social and economic effects of these measures. Each (smaller) group then elected a leader who presented the experiences and insights to the rest of the FGD participants. The FGDs were audio recorded, with all community FGDs conducted in *Luganda*. The health practitioner FGDs were conducted in English.

Key informants were purposively selected due to their involvement, expertise and influence regarding response to COVID-19 and their social and economic effects. These included individuals from government ministries, non-governmental organisations, local government, health facilities, professional bodies, and research institutions. The KIIs were conducted virtually in English via Zoom due to COVID-19 travel restrictions during the data collection period. Several measures were put in place to ensure rigour such as: referring to existing literature while designing the tools; training of research assistants before data collection; and close supervision of the data collection process.

### Data analysis

There was verbatim transcription of the audio recordings, which were in the local language (*Luganda*) for the community FGDs and household interviews. The transcripts were then verified by the research team and later translated to English. The health practitioner FGDs and KIIs which were conducted in English were auto-transcribed by a software, and the generated transcripts edited and verified by the research team. Data analysis was conducted by GBL and SN in NVivo 2020 (QSR International) guided by the thematic analysis framework using the inductive approach. Data analysis involved identifying recurring codes arising from the data with the support of a codebook. The codebook was discussed by the research team and revised accordingly. Related codes were then grouped to form sub-themes, and then themes as noted in more detail elsewhere [31]. The major themes on the social and economic effects of the COVID-19 lockdown are presented in this paper including selected quotations from participants.

### Ethical considerations

The study was approved by both Makerere University School of Public Health Research and Ethics Committee (923), and the Uganda National Council for Science and Technology (SS881ES). Permission to conduct the study was obtained from various stakeholders including the respective health offices and local leaders. All study participants provided written informed consent after the research team had explained all aspects of the study including the benefits and risks of participating. Participants understood that their participation was voluntary. All necessary measures such as assigning coded identifiers to the participants were taken to maintain their anonymity. The data was securely stored in password-protected files and only accessed by the research team. In addition, the data was only used for research purposes.

## Results

Findings from the study are presented under six themes: family disruption (domestic / gender based violence, family break-ups, loss of family housing, increased care responsibilities, and alcohol use); abuse of children’s rights (child labour, sexual abuse, and early marriage); disruption in education (home based / virtual learning, delayed educational milestones, and school drop-out rates); food insecurity (reduced access to food, reduced food varieties, and change in feeding patterns); impact on livelihoods (closure of businesses, depletion of capital and savings, agricultural losses, change o**f** job / business); and human rights violation (beating by law enforcers, and unlawful detention).

### Family disruption

#### Domestic / gender-based violence

Participants of the FGDs, household interviews, and KIIs reported that incidences of gender-based violence (GBV) increased during the lockdown. Some key informants highlighted that women were the major victims of domestic violence, mainly at the hands of their male spouses or other male household members.

*“There was a spike in gender-based violence. Very many families that we work with have reported such incidences. We talk to women about how they are at home, and the relationship between them and their husbands. Most of the women report that their husbands have become so violent, abusive and no longer listen to them these days. There was also increased domestic violence nationally from reading newspapers, and watching television. There are a lot of cases where women have been battered and some of them killed.”* Key informant 5, Non-Governmental Organization staff.

Participants cited stress due to financial challenges, the extended period of time that families spent in close proximity, and social exclusion from other family members and friends as the major drivers of conflict which led to domestic violence.

*“There were conflicts in families because the husbands were no longer working and the wives could ask for money from the husband and yet the husband did not have. In addition, husbands were spending so much time with their wives and would notice the small mistakes they otherwise would not have seen if they only came back at night. Such things would annoy a husband, he gets a cane and beats the wife.”* Participant 1, Women, FGD, Kampala.

#### Family break-ups

Participants of the community FGDs and KIIs reported that many families, especially those in urban areas, broke-up during the lockdown. They reported increased cases of men abandoning their homes and family responsibilities mainly due to financial challenges. Some family break-ups were caused by conflicts and GBV in families.

*“Lack of money and people not used to being in the same place together for long led to family disagreements and break-ups. Another thing I observed is women were used to their husbands providing for them at home but many ran away and left everything to women.”* Middle-income household, female, participant 3, Wakiso.

#### Loss of family housing

Participants of the FGDs and household interviews reported that some families could not afford their rent during the lockdown which led to evictions. They added that many men abandoned their families, leaving the women and children in rented houses. When such families could not pay rent within the grace period provided by the landlords, they were evicted and left with no housing. The participants reported that some families were forced to stay with relatives, while others stayed in abandoned churches.

*“Then there were rental problems, most of the people were evicted from rentals even when there was a directive that no tenant should be removed from their house because they were not working. However, most of the landlords didn’t listen to that, and evicted people from houses hence families had nowhere to go. Some had to go to their relatives in the villages.”* Participant 1, Female Youth FGD, Kampala.

#### Increased care responsibilities

Participants reported that women’s care responsibilities greatly increased during the lockdown. This was as a result of many women taking up financial responsibilities to support their families while continuing to engage in domestic roles. Some participants reported that women, especially those in rural areas, had more domestic responsibilities due to the larger families they had during the lockdown. They therefore had to grow more food to cater for the bigger extended family members.

*“My husband could not work during the lockdown. We almost didn’t have an income but I could not tell the children. The children’s basic needs still had to be met, so as a woman I had to make a way. That meant that I had to work to earn money but I was still the one who had to take care of these children even though their father was there. This was a story for many women in this village. Many men did not contribute to domestic roles yet women had to take up income-generating work to support their families.”* Low-income household, female, participant 3, Wakiso.

#### Alcohol use

Participants of the community FGDs and household interviews revealed that alcohol consumption, particularly among the youth and men, increased during the lockdown. This was due to the increased free time they had during the restrictions. Some participants added that alcohol was a coping mechanism for some men to deal with the different social and financial challenges such as social isolation and loss of work and income. Some participants of the community FGDs reported that since bars were closed during the lockdown, people drank alcohol at home which might have negatively influenced children in the families.

*“Alcohol consumption became worse during the lockdown. You could find young men drunk even before midday. People had a lot of free time and some drunk to forget their financial troubles for a while. We even saw children negatively influenced because their parents drank from home and some children also adopted the behaviour.”* High-income household, male, participant 2, Kampala.

### Abuse of children’s rights

#### Child labour

Participants of the community FGDs, KIIs and household interviews revealed that children’s rights were abused, and many were exploited in various ways during the lockdown. For example, there was a reported surge in child labour in various jobs such as selling foodstuffs on streets, on building construction sites, and in plastic recycling plants. Many children engaged in paid work to supplement their household income, while some were the primary income earners in their families. The participants also reported that child labour led to exploitation of children who were sometimes paid very low wages or physically injured while at work.

*“In my opinion, the lockdown worsened child labour because during that time, I would see a little girl or a little boy carrying or vending merchandise very often. Some children were as young as five years old. Very many children got engaged in child labour because their parents took advantage of them being at home.”* Key informant 5, Non Governmental Organisation staff.

#### Sexual abuse

Participants of the FGDs and KIIs reported that cases of sexual abuse especially among girls increased during the lockdown. The participants added that a large portion of the abuse was from male relatives in extended families. In addition, some girls were sexually abused by strangers while they engaged in casual jobs such as selling foodstuffs on the streets. Participants added that sexual exploitation of children led to a rise in teenage pregnancies during the lockdown especially among low-income communities.

*“The girls have been defiled, they have been raped, they have become young mothers. We have seen an influx in the number of teenage mothers, sexual exploitation, and unsafe abortions. I’m looking at premature deaths because of unsafe abortions. Unfortunately, it is the girl child who suffers the most when such lockdowns are implemented.”* Key informant 9, United Nations agency staff.

#### Early marriage

Participants of the FGDs and KIIs reported a rise in child marriages especially among girls in rural areas. They reported that children particularly teenage girls were married off by their parents or guardians in return for bride price in form of cash or property. The participants attributed this to poverty among community members and closure of schools.

*“There were cases where the girl child has been given into marriage as a result of poverty that has been developed as a result of the COVID-19 lockdown. This causes more disparity as some of those children will never have opportunities to get back to school, whereas their boy counterparts may have the opportunity to continue with their education. Child marriages have been much more in rural than urban areas.”* Key informant 8, United Nations agency staff.

### Disruption in education

#### Home-based / virtual learning

Participants of the community FGDs, KIIs and household interviews reported that suspension of schools and other learning institutions led to the introduction of virtual and home-based learning via the internet, television, radio and newspapers using government-provided learning materials. The participants reported that these modes of learning were difficult for learners who lacked the necessary materials and tools. In addition, many learners particularly in rural areas did not have access to electricity, television, computers, and internet services. It was also noted that the government reading materials did not reach those in most need, leaving learners in rural areas and poor urban communities with no formal education for an extended period of time.

*“There were education materials on television, in newspapers, and online. I think that it was all intended to ensure that the students continue learning. But at the end of the day, think about students in the rural communities who do not have access to the internet and television. Majority of the children in the villages were not benefiting from these platforms.”* Key informant 1, policy maker, Ministry of Health.

#### Delayed education milestones

Participants of the community FGDs, KIIs and household interviews reported that the closure of schools for an extended period of time led to delayed education milestones for many learners. For example, learners who were in their final years were not able to write their national examinations and had to wait for the following year. This was similar for other learners who had to progress to the next classes.

*“In terms of education, many children have been negatively affected. There were those in universities, as well as secondary and primary schools who did not sit for their final exams. All other children who had to progress to other classes are asking when they are returning to school. They ask their parents if they are going to spend seven or eight years in the university, or ten years in primary school.”* Key informant 10, Non Governmental Organization staff.

#### School drop-out rates

Participants of the community FGDs and KIIs revealed that the lockdown increased the rate of school drop-out. This was attributed to the closure of schools for extended periods of time which led to some learners losing interest in education and resorting to working to earn money. In addition, some learners, particularly girls, got married or became pregnant during the lockdown. When the schools reopened, some students did not return to school because their parents or guardians could not afford to pay school fees.

*“Children who have been making some money during lockdown found it hard to go back to school. When you tell them to resume their education, they do not see the importance anymore and others say that they are now mature enough hence do not have time to go back to school.”* Participant 10, Youth Male FGD, Kampala.

### Food insecurity

#### Reduced access to food

Participants of the community FGDs and KIIs reported that curfew and suspension of public transport hindered many families from physically accessing markets or their gardens particularly in urban settings. Some participants reported that their gardens were far and therefore needed some form of transport to access them hence faced challenges of getting food.

*“The markets were closed and on opening, many of them were far away so in that way, relying on food from markets without your own garden was difficult. Since most means of transport were suspended, getting food from markets that were far away was so hard and even if you had money, you would hardly be able to get food.”* Participant 12, Youth Women, FGD, Wakiso.

#### Reduced food variety

Study participants reported that their feeding on a variety of foods changed, and many did not have access to a balanced diet due to financial hardships during the lockdown. For this reason, many people especially in poor urban communities became reliant on a persistent diet of maize flour and beans. Indeed, many people did not have access to a variety of fruits and vegetables or other types of food. In contrast, rural communities were better able to access fruits and vegetables from their subsistence gardens.

*“It was a very difficult time when parents were struggling to find maize flour to feed their families. There is no way they could afford meat, milk or fruits to provide a balanced diet for the children.”* Key informant 8, Health Practitioner, Wakiso.

#### Change in feeding patterns

Participants of the community FGDs also reported that their feeding patterns and behaviour including the frequency of meals and portion sizes changed. Many people resorted to eating one meal a day instead of the usual two or three meals. Participants also reported that portion sizes reduced due to the scarcity of income hence food, and larger family sizes due to children being at home.

*“It is true that the feeding in families changed because those that were eating a kilogram of a certain food, started eating half a kilogram. In addition, they had to eat one meal so that they could save and survive. I am saying this because there was no money for a person for example to eat breakfast, lunch and dinner.”* Participant 4, Male FGD, Kampala.

### Impact on livelihoods

#### Closure of businesses

Participants of the community FGDs and household interviews reported that the suspension of non-essential businesses such as shopping malls and non-food markets during the lockdown greatly affected people’s sources of income and their livelihoods. Many proprietors of small and informal businesses survived on incomes earned on a daily basis and did not have savings. They were therefore affected by the sudden suspension of businesses during lockdown. Participants added that the suspension of public transport also made it difficult for people working in essential services to access their places of work. As a result, many people could not earn an income which led to financial constraints hence the inability to afford basic services such as food, medical care and housing.

*“Closure of public transport also affected the rate of earning for most of us here because many of us use public means such as commuter taxis or commercial motorcycles to go to work. Private vehicles were also not moving, and a few that were allowed to move had a restricted time, and businesses had to open from 7am to 4pm. It became so hard for us and many of our businesses collapsed.”* Participant 1, Male FGD, Wakiso.

#### Depletion of capital and savings

Participants of the community FGDs and household interviews revealed that people who were self-employed or owned small businesses were forced to spend the capital from their businesses to meet their financial needs and obligations during the lockdown. This meant that many businesses collapsed and could not reopen when the lockdown was eased. In addition, some people had to use all their personal or family savings to meet their basic financial obligations which affected their ability to pay for other needs such as school fees.

*“Most of the money that people had including their savings was used during the lockdown for household expenditure including on food and utilities. Therefore, when we were getting out of the lockdown, people had used all the money and many could not resume their businesses as they had no more capital.”* Participant 1, Youth Female FGD 1, Kampala.

#### Agricultural losses

Participants of the community FGDs especially those in rural areas reported that the restriction of travel within districts and the closure of land boarders halted their ability to sell their produce to other districts in the country or neighbouring countries. This therefore implied that perishable agricultural goods flooded the nearby markets, and farmers sold them at much lower prices. In addition, the suspension of public transport made it difficult for them to transport their produce to markets, leading to further agricultural losses. The participants revealed that many farmers resorted to giving away freely their produce to families and those in need, while others sold their produce on the farms at very low prices.

*“The farmers were severely affected by the lockdown. Milk prices fluctuated to the extent that people made high losses. They were far away from the towns and the vehicles couldn’t access the milk. Sale of eggs and chicken was also greatly affected a lot as their prices drastically dropped.”* Participant 6, Male FGD, Kampala.

#### Change of job / business

Participants reported that some community members whose jobs were suspended for extended periods of time were forced to change jobs or businesses to meet their financial obligations. For example, teachers and those who engaged in public transport shifted to working in small retail businesses or agriculture. Participants also revealed that many people completely abandoned their previous jobs, while others kept their new businesses as a supplementary source of income after the lockdown was eased. This therefore led to diversification of income to some people.

*“The jobs we used to do were suspended hence could not earn an income anymore. Therefore, there was a complete change of source of income. Even here where we are seating it was my garden, but due to COVID-19 lockdown, I decided to start-up a small retail shop. So, the lockdown brought change of livelihoods. We had some jobs we were doing which completely stopped so it brought creativity. People had to improvise and we saw another side of life we were not used to.”* High-income household, male, participant 4, Wakiso.

### Human rights violation

#### Beating by law enforcers

Participants of the community FGDs reported that human rights were grossly violated by the enforcers of lockdown measures. They reported that many community members, including pregnant women, youth and the elderly, were beaten by law enforcers when they failed to observe travel restrictions or curfew. This led to fear and panic among community members especially the men and youth who were found moving during curfew hours.

*“A man was transporting a pregnant woman to the hospital on a motorcycle without the permission from the Resident District Commissioner and he was beaten by law enforcers. The man was seriously injured by the beating and took about three months to recover.”* Low-income household, male, participant 8, Kampala.

#### Unlawful detention

The community FGDs reported that many community members who violated the lockdown measures particularly curfew were detained in police custody for more than the mandated 48 h, while others had to pay unregulated fees to the law enforcers to be released. Participants added that fear of detention aggravated other effects of the lockdown such as stress and anxiety.

*“People were very worried during the lockdown because they feared being arrested during curfew time. The law enforcers would arrest and keep us in police custody for many days, sometimes a week without any justification. Our families would not know where we were until when realised.”* Participant 6, Male FGD, Kampala.

## Discussion

This study confirmed and described the widespread and profound negative social and economic impact of the COVID-19 lockdown measures on Ugandan communities across a range of dimensions. These dimensions related to family disruption, child welfare, disruption in education, food insecurity, negative impact on livelihoods, and violation of human rights. The findings emphasize the need to mitigate such harms in response to pandemics especially in a low-income country such as Uganda. The UN framework for socio-economic response to COVID-19 [27] could be used to guide future planning for outbreak control in the country. This framework emphasizes the need to: ensure continuity of essential services; promote social protection; provide support small-scale enterprises; prioritize the most vulnerable populations; and enhance community-led response.

This study found that domestic violence, particularly violence against women, increased due to the COVID-19 lockdown measures. Financial stress, close proximity of spouses for extended periods, and social exclusion were cited as the major drivers of conflict in homes. Increased domestic violence was a recurrent theme during the COVID-19 lockdown in other studies conducted in Uganda [21, 32]. Our study also found that families broke up, with men abandoning their spouses and children mainly due to financial pressures. This in turn increased the caring responsibility and burden for women. Men abandoning their caring responsibilities could be due to the common practice of polygamous and plural relationships in Uganda [33] which meant that the COVID-19 travel restrictions impeded movement from one home to another. Our study also found that where men failed to fulfil their family financial obligations, and the women did not have a source of income prior to the lockdown, it led to loss of family housing due to failure to pay rent. Failure to pay rent has been documented in other studies conducted during the COVID-19 pandemic in Uganda [34–36] and other Sub-Saharan countries such as Kenya [37], Tanzania [38], Ghana [39] and Nigeria [40]. This highlights the gendered impact of the lockdown measures and the importance of women having some income to supplement family earnings. Families are the foundations of society and define people’s essence of life and identity [33].

In our study, child welfare, including children’s rights, were affected by the COVID-19 lockdown measures. The participants reported increased cases of child labour particularly in urban areas, sexual exploitation, and early marriages especially in rural areas. The sexual exploitation and early marriages led to teenage pregnancies. Indeed, the Ministry of Health reported a 17% increase in teenage pregnancies during the lockdown [24]. Early marriages may have been a secondary consequence of poverty and closure of schools which were direct effects of the lockdown. In addition, the higher incidence of sexual exploitation in rural areas compared to urban settings highlights the socio-economic disparities that exist between the two contexts. Moreover, children in urban areas had better access to virtual learning resources and home-based schooling materials than those in rural areas [25, 41]. Therefore, children and their parents in rural areas may have lost hope of continuing with education hence resorted to marriage. Governments should equitably plan for both urban and rural children to meet their context specific needs in order reduce their vulnerability and protect both groups especially in times of national emergencies.

This study revealed the extent of disruption to education, through the extended suspension of conventional learning, which will result in long-term negative impacts on individuals while deepening socio-economic inequalities in the country. Home-based and virtual learning used during the lockdown was inaccessible to children in rural settings and the urban poor, thus widening inequalities between the privileged and deprived groups of children [25, 41]. The challenges with virtual learning during the COVID-19 pandemic have been documented in other sub-Saharan countries [42–45]. This implies that stakeholders in the education sector including parents and children need to gradually and equitably transition towards more diverse models of learning that can be matched to different socio-economic conditions. Existing flexible paper based educational options such as the Preparation for Social Action non formal education development programme that are already existing in rural Ugandan settings [46] could be explored in future. The study also highlighted that the extended school lockdown resulted in delayed education milestones and increased school drop-out rates. This will likely have a negative impact on the children’s life trajectories and the socio-economic development of the country as a whole. Recent evidence shows that each year of schooling in Africa increases the earnings of females by 14.5% and males by 11.3% [47]. Therefore, children dropping out of school will likely have a reverse effect. The government therefore needs to plan and implement strategies to mitigate the negative long-term effects of the two-year school closure in Uganda during the pandemic.

Food insecurity emerged as a key finding in our study. Participants reported physical and financial limitations to food access during the lockdown. This therefore led to reduced food variety and changes in feeding patterns in terms of frequency and portion sizes. These findings concur with other studies carried out during the COVID-19 pandemic in Uganda [21, 26, 48–50] and other LMICs [51–55]. It is worth noting that participants in the urban setting reported more food insecurity compared to their rural counterparts. This could be due to the fact that most people in urban settings depended on daily wages to purchase food while those in rural areas mainly depended on subsistence agriculture. The suspension of non-essential businesses therefore had a substantial consequence on the earning power of most urban residents. Although the government distributed food relief packages to the urban poor, this aid was slow, insufficient, and did not reach a substantial proportion of the vulnerable population [56]. Food insecurity may have also aggravated other negative impacts of the pandemic such as sexual exploitation, domestic violence, and worsening of chronic health conditions. Food security during emergencies should therefore be systematically planned for example by stocking food reserves and establishing clear distribution channels.

The study highlighted the impact of the COVID-19 lockdown measures on livelihoods through the suspension or closure of businesses, depletion of capital and savings, agricultural losses, and change of jobs or businesses. These effects increased poverty due to significant reduction or total loss of income in communities. Similar findings related to financial hardships during the pandemic have been documented in several countries across the world [57–61]. A study estimating the economic consequences of the COVID-19 lockdown measures found that the losses due to the lockdown in Uganda were severe and may have erased the poverty eradication advancements of the past decade [34]. Most of the community participants in our study were in the informal sector and thus hardly benefited from the livelihood enhancement measures implemented by government such as the employment guarantee schemes which mainly benefited those in the formal sector [34]. The agricultural losses reported in our study affirm findings from previous studies conducted in several LMICs [62, 63]. Indeed, there was hardly any government support directly towards farmers and those in rural areas in Uganda [62]. This was in contrast to government programmes in other countries such as Zambia and Rwanda where governments provided seed and fertilizer subsidies to farmers in an effort to reduce agricultural costs [63]. It is worth noting that the informal and agricultural sectors support 34% and 75% of Uganda’s population respectively [64]. The decline in both sectors coupled with limited government support thus increased economic inequalities in Uganda. Therefore, there is need to protect and strengthen both the informal and agricultural sectors post COVID-19 lockdown to mitigate the long-term consequences posed by the pandemic and its control measures.

The study participants reported violation of human rights through beating, unlawful detention, and solicitation of unregulated fines by law enforcement officers. Men who worked in informal jobs and who were more likely to violate curfew or quarantine restrictions, were particularly affected. Findings from our research concur with other studies conducted in Uganda that found many residents to have experienced violence during the pandemic, mainly perpetrated by law enforcement officers [65, 66]. Previous studies showed that Ugandans generally feared and mistrusted law enforcement even before the pandemic [67]. However, a previous study [66] argued that the lockdown measures exacerbated the misconduct of law enforcement officers. Solicitation of unregulated fines is not surprising because Uganda ranks among the countries which high corruption among the public sector [68]. Such inappropriate conduct by law enforcement officers needs to be addressed in future pandemic response.

A major strength of this study is that it triangulated data sources from different sources (KIIs, FGDs and household interviews). In addition, a diverse range of participants at national, sub-national, and community levels participated in the study which provided a wide range of views and opinions. Data collection, particularly the FGDs, were participatory and ensured context appropriateness hence increased quality of the data. However, collecting data from only two neighbouring districts (Kampala and Wakiso) could affect the representativeness of the sample regarding Uganda as a whole hence use of the findings. Indeed, the socio-economic effects of the COVID-19 lockdown may not have been as severe in more rural districts across the country. The data was collected during a period of relatively low COVID-19 transmission between the first and second lockdowns hence the findings may have been different thereafter. In addition, having conducted the KIIs virtually could have affected the extent of engagement of the participants.

## Conclusion

Findings from the study revealed negative impacts of the COVID-19 lockdown on families such as increased gender-based violence, loss of housing, child labour, increased school drop-out rates, and delayed education milestones. In addition, livelihoods were negatively affected through depleted savings and capital, as well as human rights violation. Future response to pandemics should have in place measures to minimize the socio-economic effects of lockdown on individuals and families.

## Data Availability

All data generated or analysed during this study are included or cited in this article and its supplementary information files.

## Abbreviations

FGD: Focus group discussion
KII: Key informant interview
GBV: Gender–based violence
LMIC: Low–and middle–income–countries (LMICs)
